# Analyzing Pelvic Asymmetry by Sex and Ancestry: Insights From an Osteological Collection

**DOI:** 10.7759/cureus.59291

**Published:** 2024-04-29

**Authors:** Bryan O Ren, Robert W Goldberg, Karen D Standefer, Jason R Teplensky, Joseph P Drain, Conor F Mccarthy, John G Birch, Raymond W Liu

**Affiliations:** 1 Orthopaedic Surgery, University of Michigan, Ann Arbor, USA; 2 Paediatric Pulmonology, University Hospitals Rainbow Babies and Children’s Hospital, Cleveland, USA; 3 Orthopaedics, Scottish Rite Hospital for Children, Dallas, USA; 4 Orthopaedic Surgery, Cleveland Clinic Foundation, Cleveland, USA; 5 Orthopaedic Surgery, University of Utah, Salt Lake City, USA; 6 Orthopaedics, Walter Reed National Military Medical Center, Bethesda, USA; 7 Paediatric Orthopaedics, University Hospitals Rainbow Babies and Children’s Hospital, Cleveland, USA

**Keywords:** ethnicity, three-dimensional, osteology, sex, pelvic asymmetry

## Abstract

Introduction: Pelvic asymmetry has been noted in pelvic imaging, and might influence the development of various spinal pathologies, most notably scoliosis. There is a limited understanding of the relationship between pelvic asymmetry and sex and ancestry, and limited use of 3D modeling. The purpose of this study was to identify pelvic asymmetry and morphology differences between sex and ancestry utilizing 3D modeling on young adults in an osteological collection.

Methods: Thirty-three osteological pelvic specimens aged 18-25 years (average age 21.4 ± 2.0 years) were scanned to create virtual 3D models for analysis. Pelvic asymmetry and morphology were measured and compared across sex (male and female) and ancestry (European American and African American). Multivariate regression analysis was performed to examine the relationship between the variables measured.

Results: Multivariate regression analysis demonstrated statistically significant relationships between innominate-pelvic ring ratio and both sex (p < 0.001) and ancestry (p= 0.003) with larger ratios in male and African American specimens respectively. There was also a statistically significant relationship of greater sacral 1 coronal tilt in European American specimens (p= 0.042). There were no statistically significant differences with sex or ancestry in terms of innominate or sacral asymmetry.

Conclusion: Although there are differences in overall pelvic shape between sex and ancestry, there is no relationship between these two variables versus pelvic asymmetry in the axial or sagittal planes in young adult osteological specimens.

## Introduction

The pelvis is a nearly symmetrical structure consisting of the coccyx, fused sacral vertebrae, and bilateral innominate hip bones, each composed of three fused bones [1]. However, this aggregation introduces ample opportunity for pelvic asymmetry as an individual grows, as has been noticed clinically and described in the literature [1-3]. Pelvic asymmetry has been associated with various orthopedic pathologies, notably spinal deformities such as scoliosis [4-7]. There is uncertainty as to if the pelvic asymmetries cause the spinal deformities or vice versa. Evidence has suggested the pelvic asymmetries seen with scoliosis may not be anatomic, but rather the result of compensatory transverse plane pelvic rotation [8,9].

Traditional methods to study the human pelvis range from physically assessing osteological specimens [10,11] to using imaging such as radiographs or computed tomography (CT) scans from live patients [9]. Recently, three-dimensional (3D) modeling has been utilized for anatomical studies of the pelvis [1,2,4,9,12,13]. 3D technology has been proven to be extremely accurate in the reconstruction of osteological specimens [14]. The use of this technology can create accurate and precise replicas of a patient’s bony anatomy to help facilitate analysis.

Differences in pelvic morphology and orientation between sex [12,15] and large populations [16] have been explored in the current literature. However, the significance of asymmetry in such groups remains poorly understood as the results of these limited studies are inconclusive. In fact, these studies have shown very little or no sex and population differences in pelvic directional asymmetry [1,3,17]. Yet, high levels of absolute asymmetry have been noted [17]. Sample size was a limiting issue in one of the studies [3]. There was also variability in the methodology as some involved manually measuring osteological specimens [3,17] and others used 3D models generated from a combination of routine pelvic CT scans and postmortem specimens [1]. For the study that used 3D modeling, its use of clinical CT scans with potential clinical bias and more elderly postmortem specimens with potential changes in their pelvis with time makes it difficult to fully assess potential differences between sexes at the end of pelvic growth [1].

Therefore, the purpose of the present study was to identify potential relationships between pelvic asymmetry and sex and/or ancestry. We utilized an osteological collection of individuals who had recently completed pelvic growth (ages 18-25 years) and used 3D modeling.

## Materials and methods

Osteological pelvic samples were obtained from the Hamann-Todd Human Osteological Collection at the Cleveland Museum of Natural History in Cleveland, Ohio. This large well-documented collection contains disarticulated complete skeletons acquired from Cleveland’s unclaimed dead between 1912 and 1938. It includes contemporary documentation of each individual’s anthropometrics with photographs and radiographs, as well as notes from autopsies and dissections. Documented demographics include age at death, sex (labeled as “male” or “female”), and ancestry (labeled as “European American”, “African American”, or “Other”).

Specimens with significant bony damage (pathologic or postmortem) were excluded, as were specimens whose ancestry was labeled as “Other” as there were not enough “Other” specimens for statistical significance. Institutional review board approval was not required for this study.

**Figure 1:**
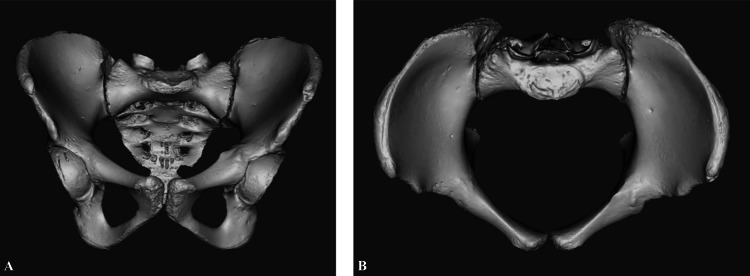
Three-dimensional assembly from a European American 18-year-old female of three individual pelvic bone models created with the ROMER scanning arm (A) Anterior-posterior view of the pelvic ring. (B) Three-dimensional assembly manipulated to show the cranial-caudal view of the pelvic ring.

Specimens were scanned using a ROMER Absolute Arm laser scanner (Hexagon Metrology, North Kingstown, RI) to create 3D models with a dimensional accuracy of ±0.040 millimeters (mm). Models were constructed and analyzed using MIMICS (Materialise NV, Leuven, Belgium), 3-Matics (Materialise NV, Leuven, Belgium), and SolidWorks software (Dassault Systèmes, Vélizy-Villacoublay, France). Each specimen’s sacrum and innominates were scanned individually, and then digitally assembled into a complete pelvis for final analysis (Figures 1A-1B).

The three separate pelvic segments (sacrum and both innominates) were reconstructed based on the sacroiliac articulation in MIMICS software using rotate and move commands and visually matching up.

**Figure 2:**
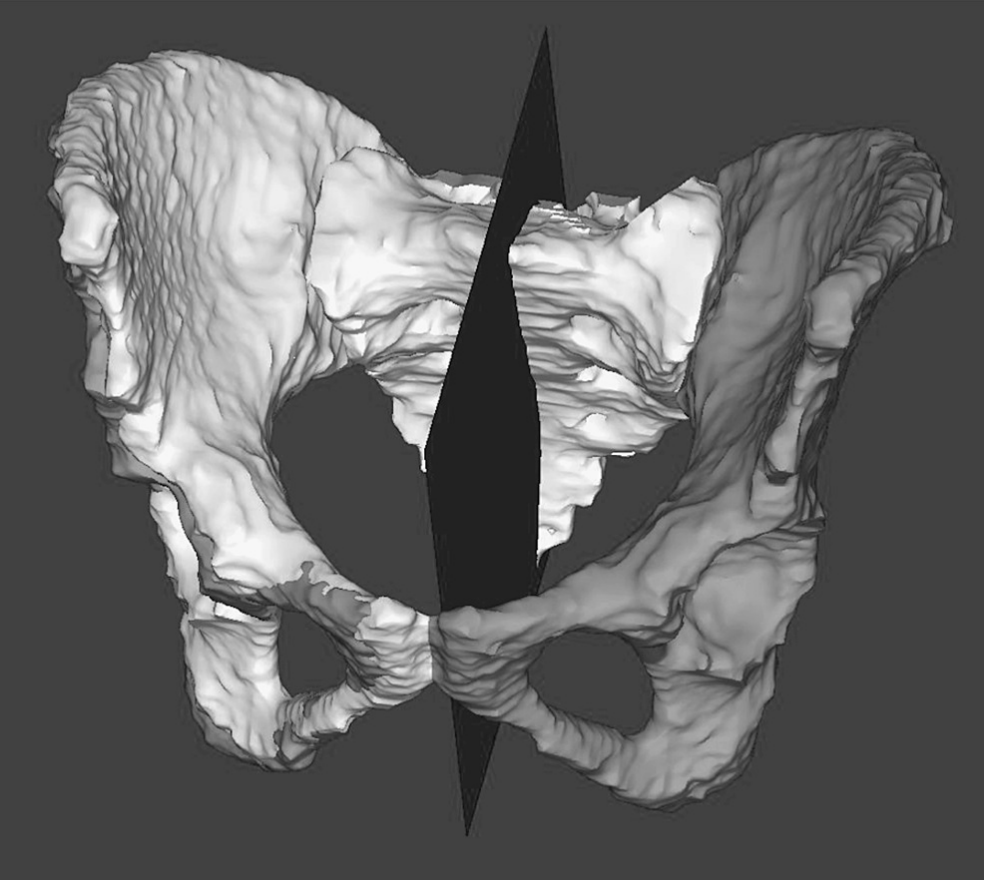
Midplane of pelvis dividing right and left sides (midway between acetabula and through pubic symphysis) The plane is used to create a mirror of the left innominate (white) onto the right innominate.

To assess pelvic and sacral midline asymmetry, the left innominate and sacrum were mirrored across the pelvic and sacral midplanes respectively, and compared to the right, noting the percentage of surface points with deviation >3 mm (Figure 2).

**Figure 3:**
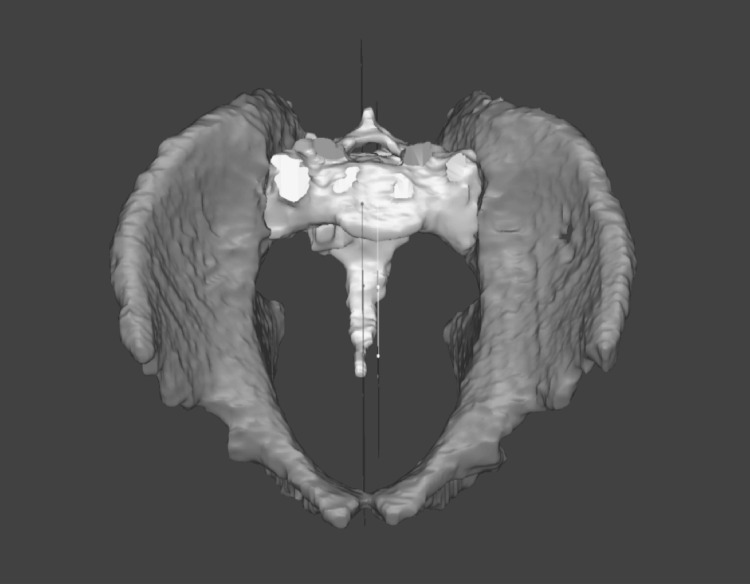
Percent S1 axial translation versus pelvic ring diameter This was determined by dividing S1 translation distance (mm) by pelvic ring diameter. The dark line represents the edge of the pelvic midplane. The white line is the edge of a parallel plane which goes through the center of S1.

The center of the pelvis was based on the center of the acetabula and pubic symphysis, which has mechanical relevance and helps distinguish if the sacrum center and pelvis center are lined up or not. Seven additional parameters were analyzed. Percent S1 axial translation versus pelvic ring diameter was determined by dividing the S1 translation distance by pelvic ring diameter (Figure 3).

**Figure 4:**
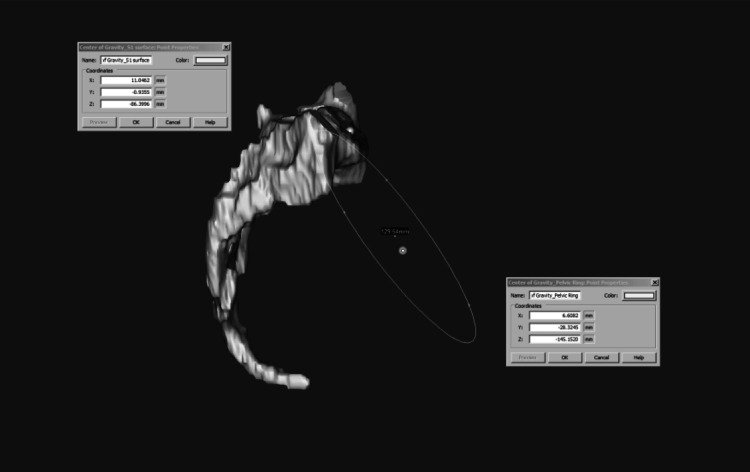
S1 anterior-posterior (sagittal) translation versus pelvic ring diameter Using the Y coordinates of the centers of the sacrum and the pelvic ring, the distance between the two centers is calculated and compared in a ratio to the pelvic ring diameter.

S1 axial rotation and percent S1 sagittal translation compared to pelvic ring diameter were determined by using the Y coordinates of the centers of the sacrum and pelvic ring; the distance between the two centers was calculated and compared in a ratio to the pelvic ring diameter (Figure 4).

**Figure 5:**
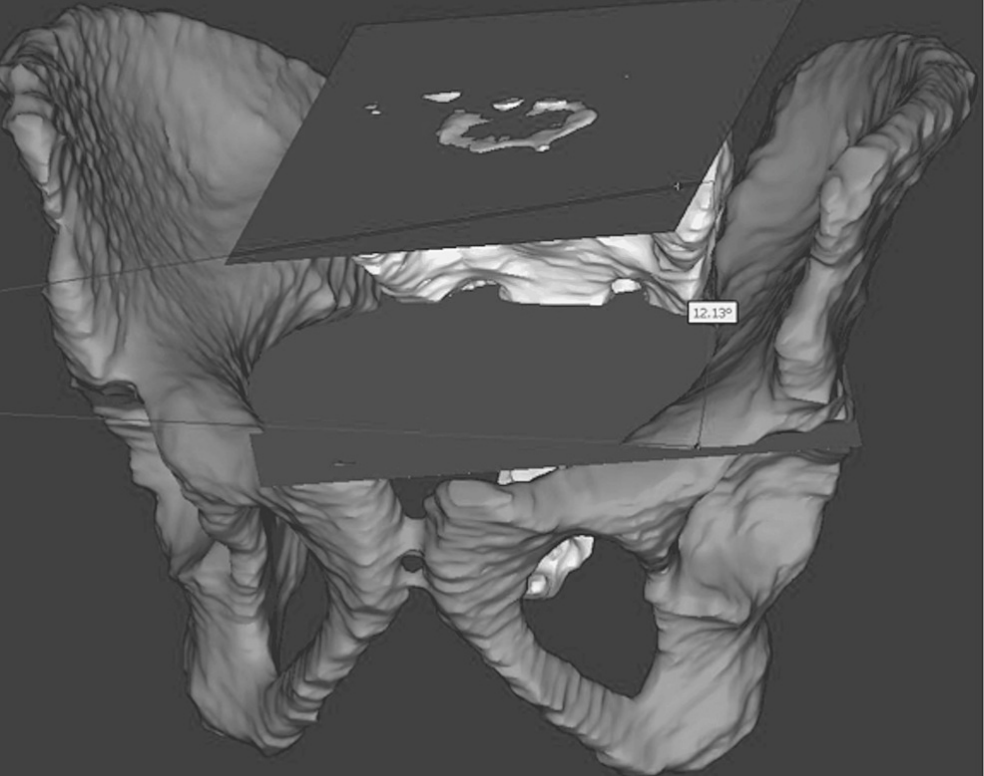
S1 coronal tilt Using the S1 fit plane (superior) and sciatic notch plane (inferior), the angle measured between these planes in the coronal plane represents coronal tilt.

S1 coronal tilt was determined by using the superior S1 fit plane and the sciatic notch fit plane and measuring the angle between these two planes in the coronal plane (Figure 5).

**Figure 6:**
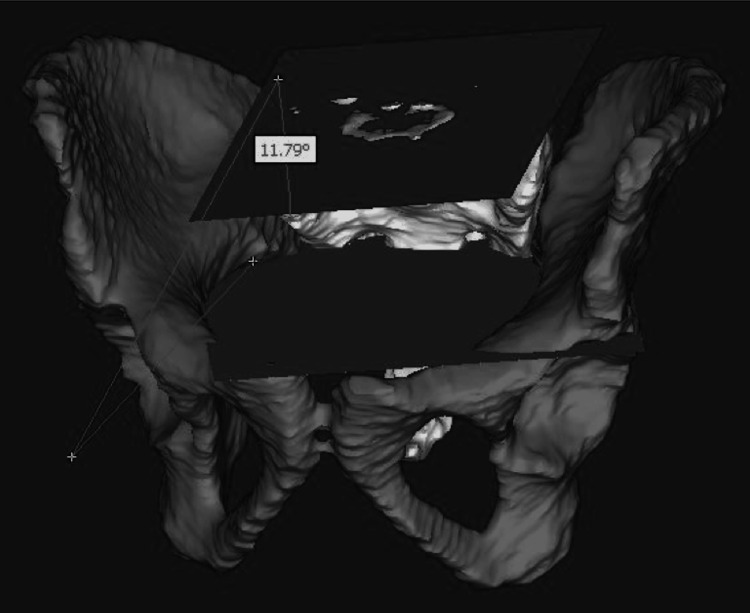
S1 sagittal tilt Using the S1 fit plane (superior) and sciatic notch plane (inferior), the angle measured between these planes in the sagittal plane represents sagittal tilt.

S1 sagittal tilt was determined using the superior S1 fit plane and the sciatic notch fit plane and measuring the angle between these two planes in the sagittal plane (Figure 6).

**Figure 7:**
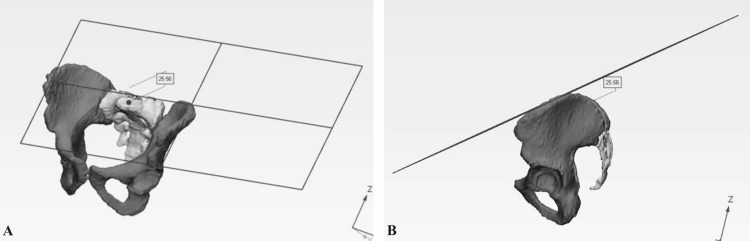
Ratio of innominate height versus pelvic ring diameter and S1 to iliac crest height versus pelvic ring diameter (A) S1 to iliac crest percent height versus pelvic ring diameter. A plane created by selecting three points on the top of the iliac crests is used to measure the distance to the center of S1 by measuring parallel to the plane. The plane and corresponding measurement were created three times for each pelvis and averaged to minimize the variance in the iliac crest. (B) Sagittal view of measurement.

The ratio of innominate height versus pelvic ring diameter and the ratio of S1 to iliac crest height versus pelvic ring diameter was determined by using a plane created by selecting three points on the top of the iliac crests to measure the distance to the center of S1 by measuring parallel to the plan (Figure 7A). The plane and corresponding measurements were created three times for each pelvis and averaged to minimize the variance in the iliac crest. A representative example of the measurement in the sagittal plane is also displayed (Figure 7B).

**Figure 8:**
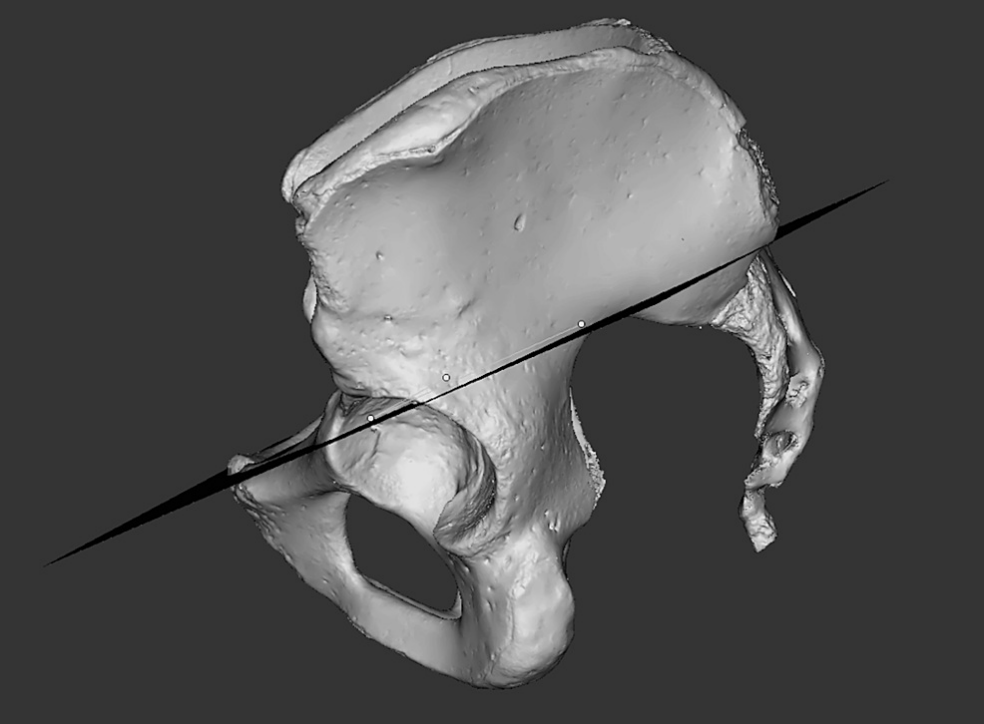
The plane used for comparing S1 tilt in coronal and sagittal planes The plane crosses through sciatic notches and pubic crest.

Coronal and sagittal S1 tilts were defined as the angles between the plane of the lumbosacral articular surface and the sciatic notch plane (Figure 8).

**Figure 9:**
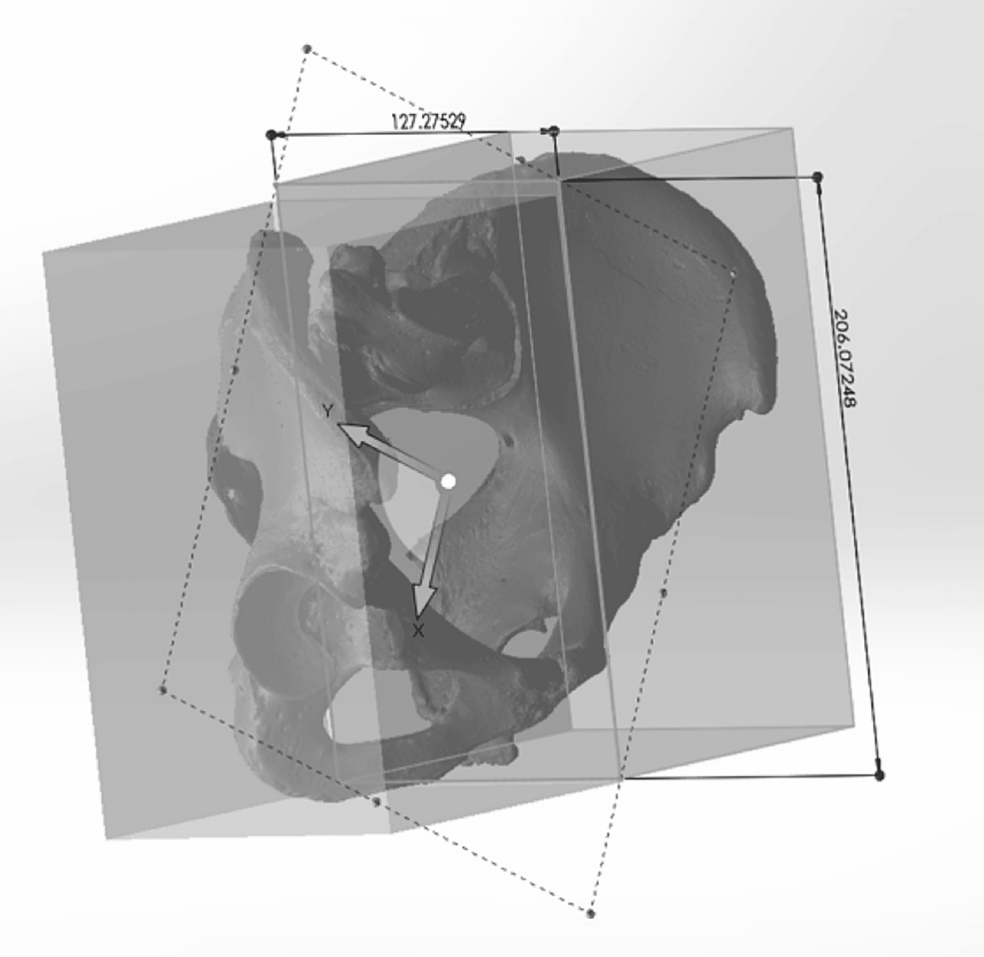
Innominate height The taller of the two sides was used.

The taller of the two innominate bones was used for the innominate height (Figure 9).

Percent S1 axial translation was defined as the distance between the edge of the pelvic midplane and the edge of a parallel plane that goes through the center of S1. S1 axial rotation was the angle between the sacrum midplane and a bisector plane that crosses through the center of S1 in the axial plane. Percent S1 sagittal translation was created by finding the distance between the Y coordinates of the centers of the sacrum and pelvic ring. S1 coronal and sagittal tilt were defined by using the S1 fit plane and sciatic notch plane and measuring the angle between the two planes in either the coronal or sagittal plane. S1 to iliac crest height was measured by creating a plane parallel to three points selected on the top of the iliac crests and measuring the distance between that plane and the center of S1. The plane and corresponding measurements were generated three times for each pelvis and averaged to minimize variance in the iliac crest.

Multiple regression analysis was performed to evaluate the association between each variable measured across the defined sex and ancestries. An alpha level of 0.05 was used to determine statistical significance. All statistics were performed using the Statistical Package for the Social Sciences (IBM SPSS Statistics for Windows, IBM Corp., Version 22.0, Armonk, NY).

## Results

Thirty-three pelvic specimens from individuals aged 18-25 (average age 21.4 ± 2.0 years) were randomly selected for study, except it was attempted to analyze an equal proportion from the documented ancestries and sex. Seventeen (52%) of the specimens were male and 20 (61%) of the specimens were African American. Further divisions were “European American male”, “African American male”, “European American female”, and “African American female” (Table 1).

**Table 1:** Characteristics of the study population The data has been represented as N, mean ± SD or N, %.

Demographics	Value
Total specimens	33
Age at death (mean ± SD, years)	21.4 ± 2.0
Sex (n, %)	
Male	17 (52%)
Female	16 (48%)
Ancestry (n, %)	
European American	13 (39%)
African American	20 (61%)

The multivariate regression analysis demonstrated statistically significant associations between innominate-pelvic ring ratio and both sex (standardized beta = -0.83, p < 0.001) and ancestry (standardized beta = 0.33, p = 0.003) with larger ratios observed in male specimens and in African American specimens (Table 2). There was also a statistically significant association of greater S1 coronal tilt in European American specimens (standardized beta = -0.34, p = 0.042).

**Table 2:** Multivariate regression analysis of variables on pelvic asymmetry p < 0.05 was considered statistically significant.

Variable	Sex: Standardized beta (Male=0, Female=1)	Sex: p-value	Ancestry: Standardized Beta (European American=0, African American=1)	Ancestry: p-value
Pelvic midline asymmetry	-0.25	0.19	-0.043	0.82
Sacral midline asymmetry	0.18	0.32	-0.039	0.82
Axial S1 translation	-0.22	0.19	0.29	0.080
Axial S1 rotation	0.26	0.15	0.30	0.086
Sagittal S1 translation	-0.092	0.63	0.18	0.34
Coronal tilt	0.12	0.49	-0.34	0.042
Sagittal tilt	-0.043	0.81	0.34	0.066
Innominate-Pelvic ring ratio	-0.83	<0.001	0.33	<0.003
S1-Iliac crest ratio	-0.32	0.089	0.094	0.60

Additionally, the average axial S1 percent translation was -0.85 ± 1.53% among European American pelvises and 0.33 ± 2.43% among African American pelvises (P = 0.098). The average S1 percent translation among male and female pelvises was 0.35 ±1.58% and 1.34 ± 1.58% (P = 0.099), respectively. The average axial S1 rotation was 0.13 ± 1.74° among European American pelvises and 1.30 ± 1.50° among African American pelvises (P = 0.058). Coronal S1 tilt was 2.51 ± 3.03° and 0.58 ± 2.96° for European American and African American pelvises, respectively (P = 0.083). There were no statistically significant differences with sex or ancestry in terms of innominate or sacral asymmetry.

## Discussion

The present study aimed to identify potential differences in pelvic asymmetry and shape between sex and ancestry. The results showed an increased ratio of innominate height versus pelvic ring diameter associated with both male sex and African American ancestry, and increased coronal S1 tilt seen in European American specimens. There were no statistically significant differences for the other parameters analyzed. Asymmetries existed from left/right size differences and/or the orientation of the three bones of the pelvis, but were not different between sex and ancestry.

These results add to the limited literature on the association between pelvic asymmetry, sex, and ancestry [1,3,17]. Most of the current studies are highly heterogeneous and there is no singular method to investigate these relationships [1]. Tobolsky et al. analyzed patterns of directional asymmetry in the pelvis and pelvic canal in an osteometric study of skeletons from five geographic regions [3]. They found the pelvis had a left bias in directional asymmetry, and the pelvic canal had a right bias in the anterior canal and a left bias in the posterior canal. The anterior canal is the anterior or ventral portion of the pelvic canal, and the posterior canal is the posterior or dorsal portion of the pelvic canal. However, the pelvis or pelvic canal did not demonstrate any sex or population differences in directional asymmetry. A similar osteometric study was performed but analyzed percentage absolute asymmetry in addition to directional asymmetry and found few sex and no population differences in percentage directional or absolute asymmetry, but high levels of absolute asymmetry in the pelvic canal [17].

Handrich et al. evaluated the asymmetry of the pelvic ring using 3D statistical modeling from pelvis CT scans in European and Asian males and females, demonstrating six distinct asymmetry patterns in the pelvic ring but no sex or population differences in directional asymmetry [1]. Their study used routine, clinical CTs for their European patients and CTs on postmortem specimens for their Asian patients (ages 20-90 years with an average age of 64 ± 15 years). They were limited in the number of Asian CTs that they could obtain. Our study evaluated European American versus African American patients and only used postmortem specimens focused on the young adult age range. We felt that this age range is important as previous studies have demonstrated changes in pelvic and acetabular bone shape with increasing adult age [18-20]. Interestingly, the asymmetry between the two sides of the human body is not limited to the pelvis and has been demonstrated in various parts such as the nasal cavity and deviated nasal septum [21].

Our study was advantageous as we used 3D modeling for analysis [3,17]. We also were able to compare the African American with the European American ancestries and obtained all our patients of different ancestries with the same methodology, as they were all osteological specimens rather than one ancestry being postmortem and another being alive [1]. Similar to the previous studies, our results did not find differences in pelvic asymmetry between sex and ancestry [1,3,17]. There were differences in regard to innominate-pelvic ring ratio and sex and ancestry, as well as the orientation of the pelvis in the coronal plane and ancestry.

Pelvic asymmetries can have implications on biomechanics and force distribution. Directional asymmetries can be, among other factors such as genetics or nutrition, the result of biologic plastic responses to imbalanced loading [3,17]. The sacrum, in particular, is believed to be highly sensitive to biomechanical influences [3]. Asymmetrical forces conveyed from the upper body through the spine influence sacral asymmetries, whereas periacetabular asymmetries are due to asymmetrical loads from the lower extremities [1]. Asymmetry of the pelvis has been shown to follow a spiral pattern, with the iliac blades turning clockwise and the pubic symphysis turning counterclockwise [2]. These variations in loading may help explain the various pathologies associated with pelvic asymmetry, ranging from chronic low back pain to adolescent idiopathic scoliosis (AIS) [5-7,22].

The results of this present study demonstrated an association between increased coronal tilt and European American ancestry. We also found that African American males have an increased ratio of innominate height versus pelvic ring diameter. Although we cannot make any conclusions on causal relationships between these variables, we believe that it is important to note and provide a framework for future studies. Hypotheses for such differences include variations in underlying genetics and/or environmental influences. Our population included adults aged 18-25 from the early 1900s, and individuals of European American versus African American ancestry likely grew up in different social, economic, and physical environments during this time period. Whether or not these differences were present at birth, developed afterward, or have some contribution from both remains up for investigation.

This study is not without limitations. Since osteological specimens were used, we were only able to analyze bony structures and did not take into account the impact of soft tissue structures. We do not believe this to be an issue because previous anatomic studies have validated the use of the same osteologic collection [10,11] and it is a known issue with other osteological studies [3,17]. The novel 3D modeling technology may present minor limitations. The process of scanning a specimen involves fully scanning one face of a specimen then flipping it over and scanning the other face, after which computer software merges the two faces together. This introduces a certain degree of assumption in the final model, though it should be considered negligible given the extreme accuracy and precision of the laser scanner (advertised dimensional accuracy of ±0.040 mm) and the power of the purpose-built software, which has also been utilized in previous studies [12,13]. We are therefore confident that these technologies are more than adequate to create digitally reconstructed pelvises and mirror images to assess asymmetry.

## Conclusions

This study presents data that suggest an increased ratio of innominate height versus pelvic ring diameter is associated with both male sex and African American ancestry, as well as increased coronal S1 tilt in European American specimens. Using 3D modeling analysis, this study strengthens the data supporting that there is no relationship between sex or ancestry versus pelvic asymmetry in the axial or sagittal planes. Overall, the results of this study should guide further research into pelvic asymmetry, sex, ancestry, and their potential relationship with other musculoskeletal pathologies.

## References

[1] Handrich K, Kamer L, Mayo K (2021). Asymmetry of the pelvic ring evaluated by CT-based 3D statistical modeling. J Anat.

[2] Boulay C, Tardieu C, Bénaim C (2006). Three-dimensional study of pelvic asymmetry on anatomical specimens and its clinical perspectives. J Anat.

[3] Tobolsky VA, Kurki HK, Stock JT (2016). Patterns of directional asymmetry in the pelvis and pelvic canal. Am J Hum Biol.

[4] Begon M, Scherrer SA, Coillard C, Rivard CH, Allard P (2015). Three-dimensional vertebral wedging and pelvic asymmetries in the early stages of adolescent idiopathic scoliosis. Spine J.

[5] Cheung KM, Cheng AC, Cheung WY, Chooi YS, Wong YW, Luk KD (2008). Right hip adduction deficit and adolescent idiopathic scoliosis. J Orthop Surg (Hong Kong).

[6] Karski T (2005). Biomechanical explanation of etiology of the so-called idiopathic scoliosis. Two etiopathological groups-important for treatment and neo-prophylaxis. Pan Arab Journal of Orthopaedics and Trauma.

[7] Saji MJ, Upadhyay SS, Leong JC (1995). Increased femoral neck-shaft angles in adolescent idiopathic scoliosis. Spine (Phila Pa 1976).

[8] Gum JL, Asher MA, Burton DC, Lai SM, Lambart LM (2007). Transverse plane pelvic rotation in adolescent idiopathic scoliosis: primary or compensatory?. Eur Spine J.

[9] Qiu XS, Zhang JJ, Yang SW (2012). Anatomical study of the pelvis in patients with adolescent idiopathic scoliosis. J Anat.

[10] Abola MV, Teplensky JR, Cooperman DR, Bauer JM, Liu RW (2018). Pelvic incidence is associated with sacral curvature, sacroiliac joint angulation, and sacral ala width. Spine (Phila Pa 1976).

[11] Abola MV, Teplensky JR, Cooperman DR, Bauer JM, Liu RW (2019). Pelvic incidence in spines with 4 and 6 lumbar vertebrae. Global Spine J.

[12] Kim JT, Shen QH, Jeon CH, Chung NS, Jeong S, Lee HD (2021). No linear correlation between pelvic incidence and acetabular orientation: retrospective observational study. Medicine (Baltimore).

[13] Li DX, Ead MS, Duke KK, Jaremko JL, Westover L (2021). Quantitative analysis of regional specific pelvic symmetry. Med Biol Eng Comput.

[14] Chae R, Sharon JD, Kournoutas I (2020). Replicating skull base anatomy with 3D technologies: a comparative study using 3D-scanned and 3D-printed models of the temporal bone. Otol Neurotol.

[15] Lewis CL, Laudicina NM, Khuu A, Loverro KL (2017). The human pelvis: variation in structure and function during gait. Anat Rec (Hoboken).

[16] Kurki HK (2007). Protection of obstetric dimensions in a small-bodied human sample. Am J Phys Anthropol.

[17] Kurki HK (2017). Bilateral asymmetry in the human pelvis. Anat Rec (Hoboken).

[18] Liu P, Yu YH, Chen CL (2013). Analysis of normal pelvis morphometry of modern Chinese southern Han female and its correlation with age [Article in Chinese]. Zhonghua Fu Chan Ke Za Zhi.

[19] Yamatani Y, Munemoto M, Ando E, Shigematsu H, Kawate K, Tanaka Y (2021). Sex differences in reference values of hip acetabular measurements using computed tomography in Japanese adults and the effect of aging on the measurement parameters. J Orthop Sci.

[20] Hegazy AA, Hegazy RA (2014). Midsagittal anatomy of lumbar lordosis in adult egyptians: MRI study. Anat Res Int.

[21] Alsubael MO, Hegazy AA (2009). Anatomical variations of the human nasal osteomeatal complex, studied by CT. Zagzig Univ Med.

[22] Yu Q, Huang H, Zhang Z (2020). The association between pelvic asymmetry and non-specific chronic low back pain as assessed by the global postural system. BMC Musculoskelet Disord.

